# Preventing early childhood transmission of hepatitis B in remote aboriginal communities in Northern Australia

**DOI:** 10.1186/s12939-022-01808-z

**Published:** 2022-12-28

**Authors:** Richard P. Sullivan, Jane Davies, Paula Binks, Melita McKinnon, Roslyn Gundjirryiir Dhurrkay, Kelly Hosking, Sarah Mariyalawuy Bukulatjpi, Stephen Locarnini, Margaret Littlejohn, Kathy Jackson, Steven Y. C. Tong, Joshua S. Davis

**Affiliations:** 1 Menzies School of Health Research, Charles Darwin University, Darwin, Northern Territory Australia; 2 Department of Infectious Diseases, Royal Darwin Hospital, Darwin, Northern Territory Australia; 3 Department of Infectious Diseases, Immunology and Sexual Health, St George and Sutherland Hospital, School of Clinical Medicine, UNSW Medicine and Health, Sydney, New South Wales Australia; 4 Population and Primary Health Care, Top End Health Service, Northern Territory Government, Darwin, Northern Territory Australia; 5 Miwatj Health Aboriginal Corporation, Nhulunbuy, Northern Territory Australia; 6 Victorian Infectious Diseases Reference Laboratory, Peter Doherty Institute for Infection and Immunity, Royal Melbourne Hospital and University of Melbourne, Melbourne, VIC Australia; 7 Victorian Infectious Disease Service, The Royal Melbourne Hospital, and Doherty Department University of Melbourne, at the Peter Doherty Institute for Infection and Immunity, Melbourne, Victoria Australia; 8 John Hunter Hospital, Newcastle, New South Wales Australia

**Keywords:** Hepatitis B, Infectious disease transmission, Prevention

## Abstract

**Background:**

Chronic hepatitis B is a public health concern in Aboriginal communities in the Northern Territory of Australia with prevalence almost four times the non-Aboriginal population. Infection is suspected to mainly occur in early life, however, the mode of transmission and vaccine effectiveness is not known in this population. WHO has set a target for hepatitis B elimination by 2030; elimination in this disproportionately affected population in Australia will require understanding of the modes of transmission and vaccine effectiveness.

**Methods:**

We conducted the study at four very remote Aboriginal communities. We approached mothers who had chronic hepatitis B and had given birth between 1988 and 2013 for consent. We obtained hepatitis B serology, immunisation and birth details from the medical record. If both mother and child had hepatitis B viral DNA detected, we performed viral whole genome sequencing.

**Results:**

We approached 45 women for consent, of whom 23 agreed to participate. We included 20 mothers and 38 of their children. Of the 20 included mothers, 5 (25%) had children who were hepatitis B immune by exposure and 3 (15%) had children with evidence of chronic hepatitis B infection at the time of assessment. Hepatitis B immunoglobulin (HBIg) had been given at birth in 29/38 (76.3, 95% CI 59.8–88.6) children, and 26 children (68.4, 95% CI 51.3–82.5) were fully vaccinated. Of the 3 children who had chronic hepatitis B, all had received HBIg at birth and two were fully vaccinated. Of the 5 who were immune by exposure, 4 had received HBIg at birth and one was fully vaccinated. Whole genome sequencing revealed one episode of definite mother to child transmission. There was also one definite case of horizontal transmission.

**Conclusions:**

Chronic hepatitis B in this context is a sensitive issue, with a high proportion of women refusing consent. Although uncommon, there is ongoing transmission of hepatitis B to Aboriginal children in remote northern Australia despite vaccination, and this is likely occurring by both vertical and horizontal routes. Prevention will require ongoing investment to overcome the many barriers experienced by this population in accessing care.

## Background

Hepatitis B is a significant public health concern in Aboriginal Australians in the Northern Territory (NT). Hepatitis B is diagnosed through a series of serological markers, the interpretation of which is given in Table 1. The seroprevalence of hepatitis B infection (i.e. HBsAg positive) was 6.08% in Aboriginal Australians compared to 1.56% in the non-Aboriginal population between 2007 and 2011 [1]. There are also higher rates of hepatitis B related liver cancer in the Aboriginal population [2].

**Table 1:** Interpretation of hepatitis B serology

Serological Result	Interpretation
Anti-HBs > 10 mIU/mL, anti-HBc negative, HBsAg negative	Immune by vaccination
Anti-HBs positive, anti-HBc positive, HBsAg negative	Immune by exposure
Anti-HBs negative, anti-HBc positive, HBsAg positive	Chronic hepatitis B infection
Anti-HBs negative, anti-HBc positive, HBsAg negative	Isolated anti-HBc positive
anti-HBs < 10 mIU/mL, anti-HBc negative, HBsAg negative	Non-immune

There are two main avenues of transmission of hepatitis B. Vertical transmission occurs from infected mothers to their newborn(s) during pregnancy or birth [3]. Horizontal transmission occurs after birth as a result of exposure of non-intact skin or mucous membranes to infected blood or body fluids, and usually occurs in early childhood when viral loads are high or through sexual transmission in later years [4, 5]. The main source of transmission of hepatitis B among Aboriginal people living in remote communities in northern Australia remains unknown but is suspected to occur either perinatally or during early childhood [6]. Acquisition of hepatitis B virus in early life is more likely to lead to chronicity and its complications such as cirrhosis and liver cancer, compared with acquisition during adulthood [7].

Rates of vertical transmission differ across endemic regions [8, 9]. These differences are thought to relate to differences in the timing of seroconversion of HBeAg, which is in turn influenced by hepatitis B genotype [10, 11]. The presence of HBeAg during pregnancy increases the risk of vertical transmission of hepatitis B from 31 to 85% without vaccination and from < 1% to approximately 9% with active and passive (HBIg) immunisation [12, 13]. There is a unique hepatitis B genotype in the Northern Territory of Australia, called genotype C4 (HBV/C4) [14]. Genotype C HBV is associated with later clearance of HBeAg and thus a high prevalence of HBeAg positive, high viral load infections during reproductive years, leading to a likely higher risk of vertical transmission [11, 14]. In the Northern Territory of Australia, universal infant hepatitis B vaccination for Aboriginal and Torres Strait Islander children was introduced in 1988 with a catch-up program for those aged 6–16 years in 1998–99 [15]. While this has led to a decrease in chronic hepatitis B prevalence to between 2 and 4% since introduction of universal birth vaccination, this does not yet meet World Health Organisation (WHO) aims for a prevalence of chronic hepatitis B to be < 0.1% in those under 5 years of age [1, 16].

The WHO has set a target for elimination of chronic hepatitis B as a public health problem by 2030 [17]. The third National Hepatitis B Strategy has recognised Aboriginal and Torres Strait Islander people and those who are pregnant as priority populations to target hepatitis B interventions [18]. Routes of transmission and vaccine effectiveness in the period of universal vaccination need to be understood to inform preventative activities to achieve the elimination targets and reduce the burden of infection experienced by these communities. The aims of this study were, in a cohort of Aboriginal children born during a period of universal vaccination to mothers with chronic hepatitis B, to determine the hepatitis B prevalence, whether vertical transmission or horizontal transmission occurred, factors associated with transmission and vaccine effectiveness.

## Methods

### Design and setting

We conducted an observational study at four very remote communities in the tropical north of the Northern Territory of Australia, between August 2016 and December 2017. The Northern Territory comprises 1,337,791 km^2^ and the four communities are classified as very remote by the Australian Statistical Geography Standard [19, 20]. Ethical approval was obtained from the Human Research Ethics Committee of the Northern Territory Department of Health and Menzies School of Health Research (HREC2015–2520).

### Study population and data collection

We approached Aboriginal mothers who had chronic hepatitis B and gave birth between 1988 and 2013 and obtained written informed consent. These mothers were found by identifying children who had been given hepatitis B immunoglobulin via the Northern Territory HBIg database. We excluded participants if they were not able to give informed consent. We used an interpreter where necessary.

We allocated a unique study number to those consenting and collected basic demographic and clinical information. We then reviewed their children’s serology. If the child had complete serology of all three seromarkers (hepatitis B surface antigen (HBsAg), hepatitis B core antibody (anti-HBc), and hepatitis B surface antibody (anti-HBs)), we did not approach them for further data but arranged appropriate follow up for clinical care. If a child’s serology was incomplete or had not been taken at an age older than 9 months, we also approached them for consent (age over 18) or assent (age under 18). We obtained blood from mothers and their children and tested for HBsAg, anti-HBc, anti-HBs and hepatitis B viral DNA viral load, at the Victorian Infectious Diseases Reference Laboratory (VIDRL).

If mother and child had a positive hepatitis B viral DNA, we performed whole genome sequencing on both viruses. We recorded data on a paper case report form, then transferred to a secure database removing identifying information for analysis.

A child was considered to be fully vaccinated if they had received four doses of vaccine including the birth dose (i.e. given within 7 days of birth) and then at least three additional doses were given at specified intervals. The specified intervals included being at least 6 weeks of age for the first dose and 6 months (168 days) for the third dose, at least 4 weeks (28 days) between the first and second dose, at least 8 weeks (56 days) between the second and third dose and at least 16 weeks (112 days) between the first and third doses [15]. If vaccines were given at too short an interval but then additional doses were given, if the above criteria were met with any of the additional doses, then the child was considered fully vaccinated.

### Statistical analysis

We calculated the proportion of mothers who gave birth to at least one child who showed evidence of virus transmission (i.e. child was either immune by exposure, has chronic hepatitis B, or had isolated anti-HBc positive), the prevalence of the hepatitis B statuses among children included in the study, and the proportion of children who had hepatitis B serology tested after 12 months of age.

We performed univariable logistic regression to determine if there were any factors associated with transmission (i.e. child was either immune by exposure, had chronic hepatitis B, or was isolated anti-HBc positive), with a plan for a multivariable model using backwards selection for any factor with a Wald *p*-value of <=0.05 on univariable analysis. Factors tested were gender, gestational age at birth, birth weight, mode of delivery, mother’s age, vaccination status and delivery of HBIg on time. All analyses were conducted using Stata version 16 (Statacorp, College Station, Texas, USA).

### Genomic sequencing

For the mother-child pairs who had sufficient viral load to have whole genome sequencing performed, we assumed mother to child transmission occurred if there was near identical sequence homology. We performed genome sequencing as previously described [21, 22]. We performed sequence analysis using SeqScape v2.7 (Applied Biosystems). We compared sequences of the hepatitis B virus isolated from the child, mother and controls using BioEdit Sequence Alignment Editor v7.2.5 [23].

## Results

### Study population

**Fig. 1:**
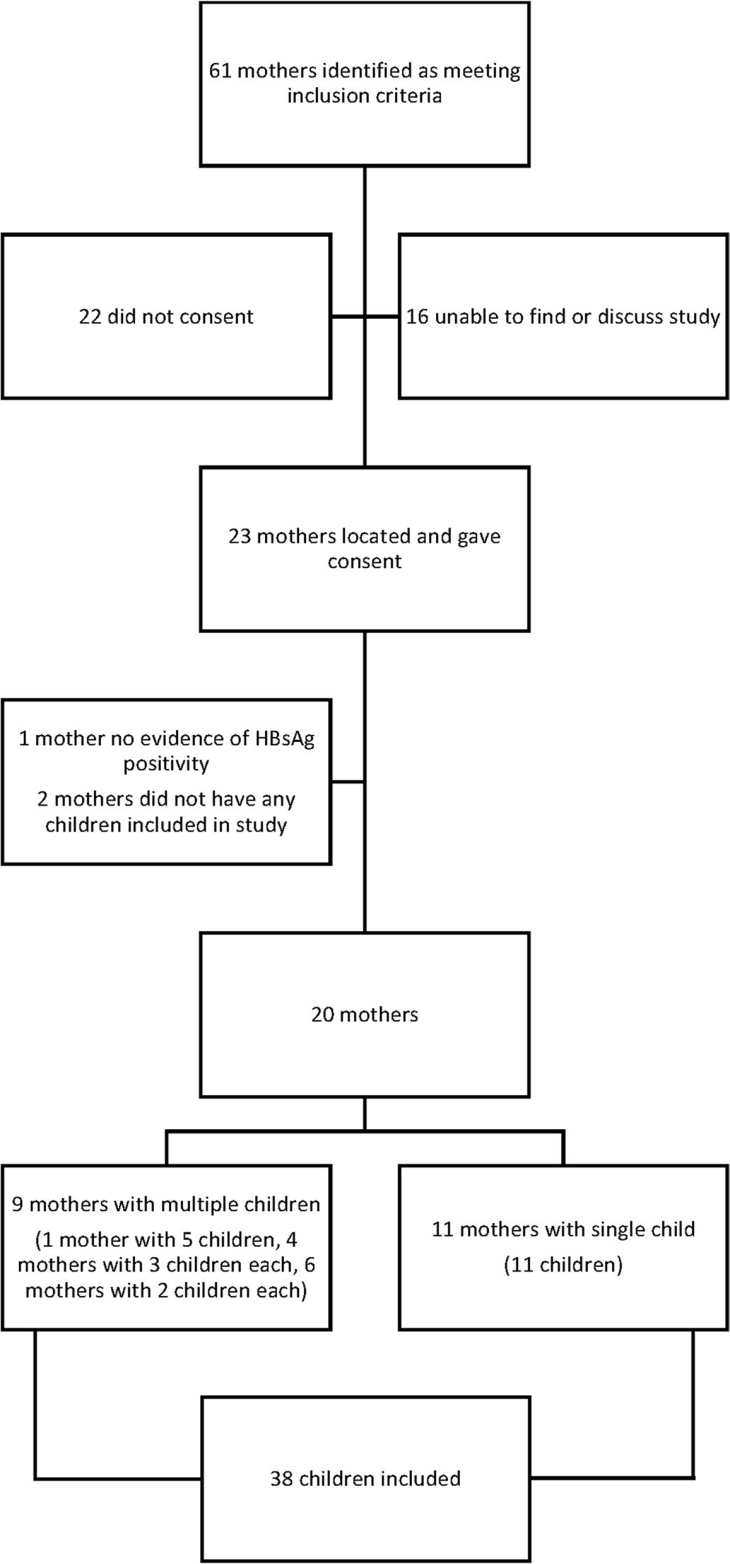
Mothers and children included in study

We recruited participants from August 2016 to December 2017. We identified 61 mothers as meeting inclusion criteria. Twenty-two did not consent and we were unable to locate or discuss the study with a further 16. Of the 23 consenting to participate, one did not have evidence of having chronic hepatitis B on subsequent review (despite their child receiving HBIg) and 2 mothers did not have any children included in the study. Twenty mothers and 38 of their children were subsequently included in the study (See Fig. 1). The baseline characteristics of mothers and children and the birth details of each child participant are given in Table 2.

**Table 2:** Baseline Characteristics of Child and Mother Participants

	Child (***n*** = 38)	Mother (***n*** = 20)
**Age (median, IQR)**	8.8 (5.7–13.0)	37.6 (34.7–40.7)
**Gender (female, %)**	22 (57.9)	20 (100)
**Community (n, %)**	Community A: 7 (18.4) Community B: 11 (29.0) Community C: 11 (29.0) Community D: 9 (23.7)	Community A: 5 (25.0) Community B: 7 (35.0) Community C: 4 (20.0) Community D: 4 (20.0)
**Gestational Age of Birth (median, IQR,** ***n*** **= 25)**	< 37 weeks: 7 (28.0) > 37 weeks: 18 (72.0)	
**Type of delivery (n = 29)**	*Uncomplicated vaginal:* 19 (65.5, 95% CI 45.7–82.1) *Elective Caesarean:* 5 (17.2, 95%CI 5.8–35.8) *Emergency Caesarean:* 5 (17.2, 95%CI 5.8–35.8)	
**Birth weight (kg, median, IQR,** ***n*** **= 28)**	2.8 kg (2.4–3.5)	
**Immunoglobulin given at birth (*n*** **= 29, %)**	25 (86.2, 95%CI 68.3–96.1)	
**Place of birth (*n*** **= 38)**	*Tertiary Hospital:* 37 (97.4, 95%CI 86.2–99.9) *Regional Hospital:* 1 (2.6, 95%CI 0.1–13.8)	

### HBIg administration

Of the 29 children where documentation of the date of HBIg administration was found, 25 (86.2, 95% CI 68.3–96.1) had received immunoglobulin within 24 hours of birth. There were nine children recorded on the register as having HBIg, but no date of administration was recorded.

### Immunisation

The majority of children had received 4 doses or more of hepatitis B vaccine (34 children, 89.5, 95%CI 75.2–97.1). Twenty-four children (63.2, 95%CI 46.0–78.2) were considered fully vaccinated. Of the 14 children not considered fully vaccinated, four did not have documentation of three doses of vaccine subsequent to the birth dose, and 10 had an appropriate number of vaccines but not at the recommended time intervals.

### Serological results

Of the 38 children, 33 (86.8, 95% CI 71.9–95.6) had complete serology either in the medical record or tested at VIDRL. Thirty-one of the 38 children already had a complete hepatitis B serology in their medical record and the median age when this was performed was 6.9 years (IQR 3.1–12.3). The hepatitis B status of all children and the characteristics of children who had chronic hepatitis B or were immune by exposure are given in Table 3.

**Table 3:** Hepatitis B status of all child participants and characteristics of children with chronic hepatitis B and children who were immune by exposure

**Hepatitis B status of child participants with complete serology**^a^								
**Status**				**Number (*n*** **= 33)**		**% (95% CI)**		
Immune by vaccination				12		36.4 (20.4–54.9)		
Immune by exposure				3		9.1 (1.9–24.3)		
Chronic hepatitis B infection				3		9.1 (1.9–24.3)		
Isolated anti-HBc positive				2		6.1 (0.7–20.2)		
Non-immune				13		39.4 (22.9–57.9)		
**Characteristics of children with chronic hepatitis B (1–3) and children who were immune by exposure (4–8)**								
**Child**	**Gender**	**Gestational Age (weeks)**	**Weight (kg)**	**Mode of delivery**	**Mother’s age at time of birth**	**Fully vaccinated**^b^	**HBIg documented as given at birth**	**Transmission**
1^c^	Female	35	2.2	Vaginal	24.9	No	Yes	Vertical (WGS)
2	Female	34	1.8	Vaginal	17.4	Yes	Yes	?
3	Male	32	1.4	Vaginal	21.9	Yes	Yes	?
4	Female	35	2.2	Vaginal	24.9	No	Yes	?
5^d^	Female	No details	No details	No details	32.6	Yes	Yes	Horizontal
6^d^	Female	38	3.5	Vaginal	27.6	No	Yes	?
7	Female	40	3.6	Vaginal	21.1	No	No	?
8	Female	39	2.6	Caesarean	32.3	No	Yes	?

^a^Of the remaining 5 without complete serology, 2 had no serology, 1 had anti-HBc and anti-HBs performed only (and was anti-HBs positive, cAb negative), 1 had anti-HBs done only (positive), and 1 had HBsAg and anti-HBs done only (HBsAg negative, sAb positive)

^b^birth dose given and three vaccines prior to 12 months of age given at appropriate intervals

^c^child’s virus isolate whole genome sequenced

^d^isolated core antibody positive

### Hepatitis B transmission

Three of the 20 included mothers (15%; 95% CI 3.2–37.9) had a child with chronic hepatitis B infection, while 5 of the 20 included mothers (25%; 95%CI 8.7–49.1) had a child who was immune by exposure. All 3 children who had chronic hepatitis B infection had received HBIg at birth and 2 were considered fully vaccinated. The child considered incompletely vaccinated had 4 doses of vaccine prior to 18 months, but not at the recommended intervals. Of the 5 who were immune by exposure, only one was considered fully vaccinated while 4 had documentation of having received HBIg at birth. Of the four with incomplete vaccination, one did not have enough hepatitis B vaccine doses for a primary immunisation course, while 3 had at least 4 doses of vaccine prior to 18 months, but not at the recommended intervals. There were two children with evidence of hepatitis B exposure and transmission who were born to the same mother: one had chronic hepatitis B and one was immune by exposure. There were no other common mothers among the children who were either immune by exposure, had chronic hepatitis B or isolated anti-HBc positive. There was no clustering in any particular community. There was evidence of horizontal transmission in one child when analysis of historical hepatitis B serology was performed (i.e. there was a negative anti-HBc result followed by a positive one).

There was one mother-child pair where there was sufficient hepatitis B DNA for whole genome sequencing and comparison. The two sequences were identified as sub-genotype C4 and had 100% identity, that is, there were no nucleotide (nt) differences detected over 3215 nt of sequence analysed. Comparison of the whole genome sequences with 61 genotype C4 sequences previously isolated from other people in the same region showed the most closely related sequence had 8 nt differences across the 3215 nt (97.75% identity). This was consistent with a transmission event between mother and child. Neither the classic G145R vaccine escape mutation nor any further known vaccine escape mutations were detected in either sequence.

There were no statistically significant factors associated with transmission on univariable analysis (i.e. child being either immune by exposure, isolated-HBc positive or having chronic hepatitis B) (Table 4). Transmission was numerically lower if the child was fully vaccinated but higher for female children and children of low birth weight but none of these observations were statistically significant.

**Table 4:** Factors associated with transmission and infection

Factor	OR for infection (95%CI) (i.e. child HBsAg + ve)	***p***-value	OR for transmission (95%CI) (i.e. child anti-HBc + ve)	***p***-value
Female gender	1.4 (0.1–17.7)	0.773	8.2 (0.9–76.2)	0.065
HBIg given at birth	–	0.855	1.1 (0.1–11.8)	0.968
Fully vaccinated	1.1 (0.1–13.5)	0.941	0.2 (0.0–1.2)	0.077
Caesarean	–	–	0.3 (0.03–2.9)	0.288
Mother’s age (per year)	0.7 (0.4–1.0)	0.078	0.9 (0.7–1.1)	0.203
Birth weight (per kg)	0.2 (0.0–1.1)	0.06	0.6 (0.2–1.7)	0.293
Gestational age (per week)	0.9 (0.7–1.1)	0.240	1.0 (0.8–1.2)	0.914

## Discussion

There is ongoing, albeit uncommon, transmission of hepatitis B in the Aboriginal population of northern Australia despite universal vaccination. Evidence of hepatitis B transmission was relatively high in the children in this study and we showed definite vertical transmission in one mother child pair by whole genome sequencing, and one episode of definite horizontal transmission. Ongoing prevention will require a multilayered approach, co-designed with the communities impacted, to minimise both these avenues of transmission in this disproportionately affected population.

Vertical transmission as demonstrated by whole genome sequencing likely reflect high rates of maternal HBeAg positivity in this region and highlights the need to have ongoing improvements in the cascade of care among pregnant women including the provision of antivirals. Aboriginal people of the Northern Territory of Australia are infected with the unique genotype C4 and compared to other genotypes, those with genotype C are less likely to lose HBeAg by gestational years [11, 14]. The presence of HBeAg correlates with hepatitis B viral load and vertical transmission is associated with both HBeAg positivity and high maternal viral loads [24–27]. Routine use of the antiviral tenofovir during late pregnancy in mothers with high viral loads to prevent mother to child transmission is now routine practice but was not during the time period for this study and so transmission rates are likely now lower [28]. The ongoing transmission shown in this study despite vaccination strategies highlights the importance of the provision of this care and antivirals during pregnancy and further research should continue to examine rates of diagnosis of hepatitis B in pregnancy, tenofovir use and enrolment into antenatal care.

To achieve hepatitis B elimination equitably across very remote Aboriginal communities a targeted and holistic approach is needed, as although the tools are available, they have been demonstrated not to be universally implemented. The “one stop liver shop” has been shown to improve the cascade of care in these communities, but there remains a significant number of individuals who are not yet diagnosed [29]. Data linkage and coding has attempted to improve identification of cases; however, many individuals do not have hepatitis B testing results [30]. A targeted strategy is likely needed for those who are pregnant to ensure hepatitis B is diagnosed and care is received. Although antenatal care is frequent in this population, first visits occur later in the pregnancy, preterm birth is common and antenatal care has been suggested to be poorly coordinated [31]. This will be even more important as return to birthing services within a community development approach is being considered [31]. The development of a culturally appropriate bilingual electronic app about hepatitis B aims to provide culturally appropriate hepatitis B care, has been translated in a number of Aboriginal languages, and has specific education about care during pregnancy to improve care during this time [32].

We demonstrated horizontal transmission in one child and high proportion of children who were immune by exposure [33]. One mechanism by which horizontal transmission could be occurring is through contact with wounds or otherwise non-intact skin in early childhood. The primary mode of hepatitis B transmission in New Zealand was thought to be direct contact through blood and skin sores [34]. In the Northern Territory, 70% of children have skin sores [35]. Continued public health response to skin health is likely to have a role in the prevention of hepatitis B transmission in our region with “The Healthy Skin Program” of the Northern Territory aiming to improve skin health [36].

The evidence of hepatitis B transmission demonstrated in our study may reflect suboptimal vaccine effectiveness. Reduced effectiveness of hepatitis B vaccine has been attributed to genetic factors, recurrent infection, passive smoking, poor nutrition, and interruption in the cold chain [37–40]. In addition, reduced effectiveness may be contributed to by the vaccine subtype mismatch between the vaccine subgenotype A2 (subtype adw2) and the circulating viral subgenotype C4 (subtype ayw3) in the Northern Territory [14, 41]. There was also a non-statistically significant trend towards transmission for those who were lower birth weight in our study, suggesting reduced vaccine effectiveness in this group.

We found delayed delivery of hepatitis B immunoglobulin at birth in some patients. There was also a high proportion of hepatitis B vaccinations occurring at intervals shorter than that which is recommended, which may be reducing vaccine effectiveness. Ten children had four vaccines prior to the age of 18 months, but not at the recommended time intervals, and transmission occurred in four of these children. Optimising timing of vaccine will be critically important in ongoing efforts to prevent transmission as post-natal care in remote health services is infrequent in this population [31].

There are encouraging results suggesting that most children had already been tested with 31 of 38 children having complete hepatitis B serology in their medical records. The median age which this occurred was 6 years old. This is still an area for practice improvement as it is recommended that children born to mothers with chronic hepatitis B have serology checked 3–12 months after completing the primary course of vaccination, which should ordinarily occur within the first 6–12 months of life [15].

The limitations of this study include that it is a retrospective study and not all birth data were available for all participants. It focused on four remote communities in the tropical north of the Northern Territory of Australia and is not necessarily generalisable to all such communities. However, we note that there was no clustering of infection in any community and although was a small sample, is likely to be similar across the Northern Territory. There was also noted difficulty in recruiting patients in this study with 22/45 (48.9%) mothers declining to participate, which also limits generalisability.

## Conclusions

In conclusion, there is evidence of limited but continued early childhood transmission of hepatitis B in Aboriginal communities in northern Australia despite vaccination and is likely occurring via both vertical and horizontal routes. Optimisation of vaccine effectiveness through improved timing of administration, the provision of antivirals in pregnancy when appropriate, investment in skin health, and ongoing effort to increase those diagnosed and in care will ensure WHO elimination goals are met and the burden of hepatitis B infection experienced by Aboriginal people living in northern Australia is reduced.

## Data Availability

As data is of a personal and confidential nature a condition of ethics approval was the raw data not be shared with any third parties.

## Abbreviations

anti-HBc: hepatitis B core antibody
anti-HBs: hepatitis B surface antibody
HBIgG: hepatitis B immunoglobulin
HBsAg: hepatitis B surface antigen
HBeAg: hepatitis B e antigen
VIDRL: Victorian Infectious Diseases Reference Laboratory
WHO: World Health Organisation
