# Correction: Preventing early childhood transmission of hepatitis B in remote Aboriginal communities in northern Australia

**DOI:** 10.1186/s12939-023-01844-3

**Published:** 2023-04-03

**Authors:** Richard P. Sullivan, Jane Davies, Paula Binks, Melita McKinnon, Roslyn Gundjirryiir Dhurrkay, Kelly Hosking, Sarah Mariyalawuy Bukulatjpi, Stephen Locarnini, Margaret Littlejohn, Kathy Jackson, Steven Y. C. Tong, Joshua S. Davis

**Affiliations:** 1 Menzies School of Health Research, Charles Darwin University, Darwin, Northern Territory Australia; 2 Department of Infectious Diseases, Royal Darwin Hospital, Darwin, Northern Territory Australia; 3 Department of Infectious Diseases, Immunology and Sexual Health, St George and Sutherland Hospital, School of Clinical Medicine, UNSW Medicine and Health, Sydney, New South Wales Australia; 4 Population and Primary Health Care, Top End Health Service, Northern Territory Government, Darwin, Northern Territory Australia; 5 Miwatj Health Aboriginal Corporation, Nhulunbuy, Northern Territory Australia; 6 Victorian Infectious Diseases Reference Laboratory, Peter Doherty Institute for Infection and Immunity, Royal Melbourne Hospital and University of Melbourne, Melbourne, VIC Australia; 7 Victorian Infectious Disease Service, The Royal Melbourne Hospital, and Doherty Department University of Melbourne, at the Peter Doherty Institute for Infection and Immunity, Melbourne, Victoria Australia; 8 John Hunter Hospital, Newcastle, New South Wales Australia

**Correction: Int J Equity Health 21, 186 (2022)**

**https://doi.org/10.1186/s12939-022-01808-z**

After publication of this article [1], the authors reported that the title was incorrectly given as ‘Preventing early childhood transmission of hepatitis B in remote aboriginal communities in Northern Australia' but should have been ‘Preventing early childhood transmission of hepatitis B in remote Aboriginal communities in northern Australia'.

The original article [1] has been corrected.
